# Outcome Prediction of Consciousness Disorders in the Acute Stage Based on a Complementary Motor Behavioural Tool

**DOI:** 10.1371/journal.pone.0156882

**Published:** 2016-06-30

**Authors:** Jean-Michel Pignat, Etienne Mauron, Jane Jöhr, Charlotte Gilart de Keranflec'h, Dimitri Van De Ville, Maria Giulia Preti, Djalel E. Meskaldji, Volker Hömberg, Steven Laureys, Bogdan Draganski, Richard Frackowiak, Karin Diserens

**Affiliations:** 1 Acute Neurorehabilitation Unit, Department of Clinical Neurosciences, University Hospital of Lausanne, Lausanne, Switzerland; 2 Department of Clinical Neurosciences, University Hospital of Lausanne, Lausanne, Switzerland; 3 Faculty of Medicine, Lausanne University, Lausanne, Switzerland; 4 Coma Science Group, University of Liege, Liege, Belgium; 5 Institute of Bioengineering, Ecole Polytechnique Fédérale de Lausanne, Lausanne, Switzerland; 6 Faculty of Medicine, University of Geneva, Geneva, Switzerland; 7 Department of Neurology, SRH-Gesundheitszentrum, Bad Wimpfen, Germany; University of Pennsylvania, UNITED STATES

## Abstract

**Introduction:**

Attaining an accurate diagnosis in the acute phase for severely brain-damaged patients presenting Disorders of Consciousness (DOC) is crucial for prognostic validity; such a diagnosis determines further medical management, in terms of therapeutic choices and end-of-life decisions. However, DOC evaluation based on validated scales, such as the Revised Coma Recovery Scale (CRS-R), can lead to an underestimation of consciousness and to frequent misdiagnoses particularly in cases of cognitive motor dissociation due to other aetiologies. The purpose of this study is to determine the clinical signs that lead to a more accurate consciousness assessment allowing more reliable outcome prediction.

**Methods:**

From the Unit of Acute Neurorehabilitation (University Hospital, Lausanne, Switzerland) between 2011 and 2014, we enrolled 33 DOC patients with a DOC diagnosis according to the CRS-R that had been established within 28 days of brain damage. The first CRS-R assessment established the initial diagnosis of Unresponsive Wakefulness Syndrome (UWS) in 20 patients and a Minimally Consciousness State (MCS) in the remaining13 patients. We clinically evaluated the patients over time using the CRS-R scale and concurrently from the beginning with complementary clinical items of a new observational Motor Behaviour Tool (MBT). Primary endpoint was outcome at unit discharge distinguishing two main classes of patients (DOC patients having emerged from DOC and those remaining in DOC) and 6 subclasses detailing the outcome of UWS and MCS patients, respectively. Based on CRS-R and MBT scores assessed separately and jointly, statistical testing was performed in the acute phase using a non-parametric Mann-Whitney U test; longitudinal CRS-R data were modelled with a Generalized Linear Model.

**Results:**

Fifty-five per cent of the UWS patients and 77% of the MCS patients had emerged from DOC. First, statistical prediction of the first CRS-R scores did not permit outcome differentiation between classes; longitudinal regression modelling of the CRS-R data identified distinct outcome evolution, but not earlier than 19 days. Second, the MBT yielded a significant outcome predictability in the acute phase (p<0.02, sensitivity>0.81). Third, a statistical comparison of the CRS-R subscales weighted by MBT became significantly predictive for DOC outcome (p<0.02).

**Discussion:**

The association of MBT and CRS-R scoring improves significantly the evaluation of consciousness and the predictability of outcome in the acute phase. Subtle motor behaviour assessment provides accurate insight into the amount and the content of consciousness even in the case of cognitive motor dissociation.

## Introduction

Attaining an accurate diagnosis remains one of the most challenging tasks with patients with Disorders of Consciousness (DOC) in the acute phase; an accurate diagnosis is crucial for prognosis validity because it influences medical management in terms of therapeutic choices and end-of-life decisions. Disorders of consciousness include a wide range of medical conditions in which patients present a global inability to interact with their environment in terms of wakefulness and awareness [1]. Among the broad nosology of DOC [2], three main ascending levels have been clinically identified according to behavioural criteria: Coma [3], Unresponsive Wakefulness Syndrome (UWS) [4], and a Minimally Conscious State (MCS) [5]. After an acute brain injury (BI), nearly 20% of patients suffer from DOC [6]; the majority of these patients fall into a coma, which is associated with a mortality rate of 40% within 6 hours in cases of traumatic BI (TBI) [7] and within 5 days in cases of non-traumatic BI (NTBI) [8]. Coma survivors then enter into a gradual process of consciousness recovery by evolving first into UWS and then into MCS until they emerge from DOC. However, the natural progression of recovery varies substantially across patients; between 10% [9, 10] and 30% [11] of coma survivors remain in UWS after four weeks. Moreover, the speed of recovery and the type of injury influence the level of recovery. Approximately 50% of patients experiencing UWS at least one month after BI remain in this state one-year after their TBI [9, 10, 12, 13]; this fraction increases to 85% post-NTBI [9, 10, 12–14]. With regard to the same time conditions, 23% [15, 16] and 43% of MCS [15] patients do not regain consciousness 12 months after TBI and NTBI, respectively.

Therefore, the most pressing clinical issue in the acute phase is to determine whether the diagnosis of these patients as being in UWS or MCS is correct and whether it is transient and a precursor of recovery. Although neuroimaging based on magnetic resonance imaging (MRI) or positron emission tomography [17–22] and neurophysiological studies [23–28] have led to major changes in the nosology of patients with DOC, the current standard for evaluating such states remains clinical examination. The JFK Coma Recovery Scale-Revised (CRS-R) [29] is currently the best instrument for DOC evaluation [30] [31]; this instrument seeks signs of conscious awareness using several subscales based on motor and verbal behaviour. Despite these advances in the nosology of DOC, the prevalence of misdiagnoses is as high as 43% in the post-acute and chronic phases [32] [33] and outcome predictability remains quite poor [30]. Further improvements in clinical assessment are accordingly necessary, especially when considered in the acute stage. Bias in diagnostic assessment in the acute phase may arise from erroneous quantification and interpretation of behavioural responses. Indeed, underlying motor, verbal or drive deficits [34] [35] may partially or completely inhibit intentional responses. Otherwise, the CRS-R scales require stringent criteria to define a certain level of consciousness, which in turn may lead to clinical underestimation of the content and amount of consciousness, if those criteria are not fulfilled. Therefore, exploring the broadest range of motor behavioural responses may result in a more accurate consciousness assessment that enables a more reliable outcome prediction. This longitudinal study presents the results of a combined CRS-R assessment with a new set of clinical tests measuring various motor signs in the acute phase.

## Materials and Methods

### Participants and study design

We enrolled 33 patients (20 males and 13 females) among 65 patients who were admitted to our Unit of Acute Neuro-Rehabilitation (Department of Clinical Neurosciences at the University Hospital of Lausanne, Switzerland) between October 2011 and June 2014 for acute neuro-rehabilitation.

The inclusion criteria included a diagnosis of UWS or MCS (according to CRS-R criteria [29], supported by the Multi-Society Task Force Report [4] [36] and the Aspen criteria [5]) within 28 days of brain damage; we assumed a short cut-off time to avoid patients with prolonged DOC who might have biased the outcome assessment due to their lower probability of recovering [29, 37].

The exclusion criteria included a) current neuromuscular function blockers or sedation; b) a premorbid history of developmental, psychiatric or neurological illness resulting in documented functional disabilities at the time of damage; c) persistent acute illness or progressive systemic or neurological disease; d) fewer than three complete clinical assessments with the CRS-R throughout the study.

The first CRS-R evaluation was completed using our own set of clinical tests or observations referred to as the “Motor Behaviour Tool” (MBT), which will be described below.

We collected general patient information: Age, localisation of brain lesions from routine neuro-imaging, brain damage aetiology, total number of CRS-R performed, rehabilitation duration and delay between occurrence of brain lesion and the first CRS-R/MBT assessment, as between occurrence of brain lesion and the last CRS-R score.

All of the included patients received a standardised intensive programme of rehabilitation, including physical, occupational, neuropsychological and speech therapies totalling at least 3 hours per day.

The local Lausanne Ethics Committee approved this study (142–09) and the legal surrogates of all of the participants provided written informed consent.

### Procedure

We designed a two-stage procedure.

First, the enrolled patients underwent at least three successive CRS-R assessments with the aim of ensuring a weekly evaluation. The first assessment established the initial diagnosis of UWS or MCS [29] [30], and the following CRS-R scores determined the DOC outcome; motor (score = 6 of CRS-R subscale 3) or communication (score = 2 of CRS-R subscale 6) recovery defined emergence from DOC [29]. We divided the patients into two primary classes: Patients recovering consciousness with functional object use and/or functional communication (class 1) and patients remaining in DOC (class 2). We made the same subdivision for UWS and MCS, which led to six additional subclasses: (a) UWS and (b) MCS patients evolving to non-DOC, UWS patients (c) remaining in UWS or (d) evolving to MCS, and MCS patients (e) worsening to UWS or (f) remaining in MCS. We performed nine outcome comparisons: One global comparison between both main classes 1 and 2, and eight additional subclasses’ comparisons encompassing four comparisons within the UWS patients and within the MCS patients, respectively. As a result, for the UWS patients, we compared subclass (a) with subclass (c), subclass (a) with subclass (d), subclass (c) with subclass (d), and subclass (a) with subclasses (c) and (d) together, which yielded a comparison between UWS patients emerging from DOC and those remaining in DOC. Likewise for the MCS patients, we compared subclass (b) with subclass (e), subclass (b) with subclass (f), subclass (e) with subclass (f), and subclass (b) with subclasses (e) and (f) together, which yielded a comparison between MCS patients emerging from DOC and those remaining in DOC. We analysed the first five CRS-R subscales because their scores defined the criteria for UWS and MCS, while the total score and the arousal subscore do not.

Second, we completed the CRS-R evaluation using the MBT evaluation, which was performed twice: At the first clinical evaluation with CRS-R, and two days later for confirmation. The CRS-R requires stringent response criteria for each subscore to assign a behavioural level and particularly to move from a low level of motor reflex behaviour (subscores 1–4) or absent verbal response (subscore 5) to the next level, which integrates the first clinical signs of consciousness. The application of stringent criteria means that insufficient but true signs of consciousness may not be quoted as an indication of consciousness, but rather as a reflex behaviour. Moreover, concomitant deficits or pathologies, including neuromyopathy, aphasia, or severe disorders of drive and motivation, additionally hamper motor or verbal responses [34]. Hence, both conditions lower the CRS-R score, leading to misdiagnoses of DOC.

We designed the MBT to complement the description of limb, facial, ocular and oral motricity, and verbal behaviour. This tool consists of a set of 10 clinical items that explore different positive motor signs (items 1 to 5) that the CRS-R scheme may overlook, medical conditions (items 6 and 7) that may hide conscious signs (negative signs) and various reflex responses (items 8–10) (Table 1). The kinematics of non-reflex or intentional movement were defined in opposition to the kinematics of spinal motor reflexes and the nociceptive withdrawal reflexes triggered at rest; for example, the maximal elicitation of the mechanical reflex response in the upper limb due to a nociceptive stimulation applied on the index finger, consists of wrist adduction (frontal plane), elbow flexion (sagittal plane), and shoulder anteflexion (sagittal plane) occurring in two planes [38]. The first item looks for any limb movement whose kinematics differ from the motor reflex response in terms of orientation planes and the type of elicited muscles; such movements may be spontaneous or induced by a sensitive stimulation. The second item explores any isolated ocular movements that may be related to a hint of fixation or pursuit. The third item looks for hints of isolated intentional movements elicited by verbal command; reproducibility is typically lacking due to concomitant disorders, such as perseveration or drive disorders. The fourth item looks for any hint of a grimace induced by a noxious stimulus. The fifth item explores any motor behaviour that can be enhanced by a motivational context, such hearing a familiar voices or engaging in outdoor therapy. The sixth and seventh items take into account concomitant pathologies, including cranial nerve palsy, myopathies, and severe disorders of drive, which may hide facial motion or vocalisation, respectively. The last three items looks for increased motor reflexes triggered by simple stimulation (limb and oral, items 8 and 10 respectively) and for roving eyes.

**Table 1:** Description of the items of the Motor Behavioural Tool.

	MOTOR BEHAVIOUR TOOL (MBT)	Yes	No
1	Observation of non-reflex movements (hypokinetic, spontaneous, or induced by a sensitive or a nociceptive stimulus)		
2	Observation of isolated ocular movements (fixation with low reproducibility, pursuit with signs of perseveration)		
3	Presence of intentional movements to command but with low reproducibility (due to signs of perseveration, to disorders of attention or to aphasia)		
4	Observation of facial movement in response to noxious stimulation		
5	Observation of increased motor behaviours in a motivational context (familiar voices, outdoor therapy, enjoyable stimulation)		
6	Facial akinasia in the context of concomitant causal pathologies (cranial nerve palsy, cranial or peripheral neuromyopathies, and disorders of drive and motivation linked with strategic lesions based on neuroimaging or on electromyographic data)		
7	In case of extubation or tracheostomy with speaking valve: absence of vocalization in the context of concomitant causal pathology (cranial nerve palsy, cranial or peripheral neuromyopathies, and disorders of drive and motivation, linked with strategic lesions based on neuroimaging or on electromyographic data)		
8	Abnormal motor or neurovegetative reflex induced by stimulation		
9	Signs of roving eyes or absence of oculocephalic reflex		
10	Increased oral reflex		

The MBT uses a non-cumulative binary scoring system with 1 and 0 indicating the presence or absence of a clinical item, respectively. Since the MBT complements CRS-R measurements, we expected the MBT to weight positively the score of the first five CRS-R subscales, when the latter are quoted at the reflex level for the motor responses (scores 1, 1, 2, and 1 for the auditory, visual, motor, and oromotor functions, respectively) and at 0 (none) for the communication function. When at least one positive MBT sign (items 1–5) is present, the principle is to increment progressively (step of 0.1) the value of the CRS-R subscale when quoted at the reflex level, until attaining the next score that highlights the first clinical signs of consciousness. For example, if the visual function scale is set to the reflex level of 1, then this value is progressively increased (1.1, 1.2, 1.3,…, 1.9) when the first positive MBT sign is observed. We hypothesise that MBT items 1, 3 and 5 increase all five CRS-R subscales; item 2 increases the auditory and visual function scales, and item 4 increases the oromotor and motor function scales (Table 2). We performed statistical analysis on each increment to identify the critical point discriminating the different outcome of classes and subclasses.

**Table 2:** Increase of the CRS-R subscores in presence of at least one positive MBT item.

	Auditory function scale	Visual function scale	Motor function scale	Oromotor function scale	Communication scale
6			Functional object use		
5		Object recognition	Automatic motor response		
4	Consistent movement to command	Object localization: reaching	Object manipulation		
3	Reproducible movement to command	Pursuit eye movements	Localization to noxious stimulation	Intelligible verbalization	
CRS-R Increase			⇧		
			MBT items 1-3-4-5		
2	Localization to sound	Fixation	Flexion withdrawal	Vocalization/oral movement	Functional: accurate
CRS-R Increase	⇧	⇧		⇧	
	MBT items 1-2-3-5	MBT items 1-2-3-5		MBT items 1-3-4-5	
1	Auditory startle	Visual startle	Abnormal posturing	Oral reflexive movement	Non-functional: intentional
CRS-R Increase					⇧
					MBT items 1-3-5
0	None	None	None/flaccid	None	None

Two members of our research team, which includes a physician, a neuropsychologist, and a nurse, conducted the CRS-R and MBT assessment using video recordings.

Since patients share the same kind of structural brain lesions with different DOCs, or present different lesion localisations within the same consciousness disorder, structural MRI cannot differentiate states of consciousness [17]. We performed MRI morphometric analysis on our data to confirm this statement.

### Statistical analysis

We performed three methodological steps in our statistical analysis.

The first step consisted of analysing the CRS-R data. First, we performed the statistical prediction of DOC outcome using the first CRS-R subscores. Our statistical testing was based on a non-parametric Mann-Whitney U test because of the multinomial distribution of the CRS-R data. Second, since the CRS-R assessments were made across patients at different time points and with variable frequencies, we fit the longitudinal CRS-R data of each patient with a Generalised Linear Model (GLM) to obtain valuable temporal information regarding the DOC evolution of all classes/subclasses. The model regressors for the GLM consisted of four polynomials with progressive degrees ranging from 0–3 (constant, linear, quadratic and cubic polynomials) defining a polynomial regression model in one variable, referred to as a cubic model. We orthogonalised the polynomials such that the measured CRS-R variable could be projected into a canonical space spanned by the regressors. We then used the polynomial regressors according to a combinatorial process. As a normality test, we adopted the Lilliefors test [39], which revealed that the calculated regression coefficients followed a Gaussian distribution. We applied a one-sample two-tailed *t*-test on the regression coefficients, and then we calculated the temporal mean curve with its standard error for each class and subclass.

In the second step, we statistically predicted the DOC outcome from the MBT values also using a non-parametric Mann-Whitney U test because of the Bernoulli distribution of the binary MBT data. We computed sensitivity, specificity, and Yule’s Q coefficient to test the performance of the statistical comparison.

The third statistical step consisted of assessing the outcome prediction on each increment of the CRS-R subscores enhanced by the MBT to identify a possible critical point discriminating the DOC outcome. The multinomial distribution of the CRS-R scores justified the application of a non-parametric Mann-Whitney U test.

Our study focused on a primary endpoint—the recovery prediction from DOC at discharge from our unit—and a secondary endpoint—the combined endpoint referring to functional walking ability and the likelihood of returning home.

We performed all of the statistical comparisons for each step between classes 1 (patients recovering consciousness with functional object use and/or functional communication) and 2 (patients remaining in DOC) and between the following subclasses: UWS patients evolving to non-DOC compared with UWS patients remaining in UWS and/or evolving to MCS; MCS patients evolving to non-DOC compared with MCS patients worsening to UWS and/or remaining in MCS. We applied the Bonferroni-Holm multiple comparison procedure to control the family-wise error rate. We performed the statistical testing in Matlab 12.0 (MathWorks, Natick, Massachusetts, USA).

To assess the anatomical differences between classes 1 and 2, we performed MRI voxel-based morphometry (VBM) [40] using the fast diffeomorphic image registration algorithm [41] and the statistical approach of statistical parametric mapping (SPM) (http://www.fil.ion.ucl.ac.uk/spm/).

For multiple comparisons correction, we applied a false discovery rate (FDR) control method in VBM. We used Matlab 12.0 for the algorithm implementation of statistical testing and SPM.

## Results

In Table 3, we present detailed information about individual patient demographics (i.e., age, localization of brain lesions, BI aetiology, total number of CRS-R performed until discharge from the unit, rehabilitation duration and the delay between the occurrence of brain lesions and the first CRS-R/MBT assessment, as between the occurrence of brain lesions and the last CRS-R score).

**Table 3:** Information about individual patient demographics. CRS-R: Coma Recovery Scale-Revised; DOC: disorders of consciousness; UWS: unresponsive wakefulness syndrome; MCS: minimally conscious state; TBI: traumatic brain injury; IS: ischemic stroke; HS: haemorrhagic stroke; RA: ruptured aneurysm; IVH: intra-ventricular haemorrhage; DAI: diffuse axonal injury; F: frontal; T: temporal; P: parietal; O: occipital; BG: basal ganglia;Mes: mesencephalon; r: right; l: left; b: bilateral.

Patient No.	Age (years)	Diagnosis	Brain lesion localisation	Etiology	Delay insult first CRS-R (days)	Numbers of CRS-R	Delay insult—last CRS-R (days)	Rehabilitation duration (days)	Outcome	Return home	Walk
1	20	UWS	bF, rT, rP, rBG	TBI	19	4	54	21	Non DOC	Yes	Yes
2	76	UWS	rP, rO	IS	15	4	70	59	Non DOC	No	No
3	51	MCS	lF, bT, bP	TBI	5	3	41	16	MCS	No	Yes
4	57	UWS	rF, rT	HS (RA)	27	4	61	21	MCS	Yes	Yes
5	48	MCS	Pons	HS	9	3	94	55	Non DOC	No	No
6	25	MCS	rF, bT, rP	TBI	13	3	94	7	Non DOC	Yes	Yes
7	53	MCS	bT, lP	TBI	9	4	25	19	Non DOC	Yes	Yes
8	67	UWS	lF, lP, rO	IS (RA)	18	4	41	48	Non DOC	Yes	Yes
9	53	MCS	rBG	Inf	12	6	98	21	Non DOC	Yes	Yes
10	41	UWS	rF, rT, rP, rBG	HS	5	7	34	20	Non DOC	No	No
11	24	UWS	bF, lBG, DAI	TBI	9	7	52	35	MCS	Yes	No
12	65	MCS	lBG	Inf	12	6	60	33	MCS	NA	NA
13	68	MCS	lF, lP	HS	6	3	39	37	Non DOC	No	No
14	65	MCS	bF	TBI, IS	7	3	26	14	Non DOC	Yes	Yes
15	18	UWS	bF, bT, lBG, Mes	TBI	15	6	69	41	MCS	Yes	Yes
16	55	UWS	rT, rP	HS	14	3	54	20	Non DOC	Yes	Yes
17	24	MCS	rT	TBI	4	3	26	8	Non DOC	Yes	Yes
18	73	UWS	Pons	IS, IVH	7	9	56	43	Non DOC	Yes	NA
19	68	UWS	bF	TBI	2	3	24	15	Non DOC	Yes	Yes
20	37	UWS	bF, bT, bP, bO, bBG	Anoxia	8	8	65	24	UWS	No	No
21	35	UWS	bF, bT, lP	TBI	18	7	59	33	UWS	No	No
22	49	UWS	bBG, Mes, Pons	IS	27	6	65	42	MCS	No	No
23	20	MCS	bF, bT, bP, DAI	TBI	7	3	33	13	Non DOC	Yes	Yes
24	66	MCS	bT	TBI	9	3	25	13	Non DOC	Yes	Yes
25	24	UWS	bBG, Mes, Pons	TBI (PO	5	9	48	36	MCS	No	No
26	59	UWS	bF	TBI	11	3	31	14	Non DOC	Yes	Yes
27	22	UWS	lF, lT, lP	TBI	11	8	31	8	UWS	No	No
28	53	UWS	rF, rT	HS (RA)	8	9	64	41	UWS	No	No
29	63	UWS	bF, bP	IS (RA)	18	10	71	48	Non DOC	Yes	Yes
30	62	UWS	rF, rT, bP, bO	TBI (Op)	5	9	75	33	Non DOC	Yes	No
31	55	MCS	bF, bP, bBG	HS	1	7	53	36	Non DOC	Yes	Yes
32	42	UWS	lBG, DAI	TBI	16	5	40	22	Non DOC	Yes	Yes
33	43	MCS	lF, bBG, Mes	IS	17	3	44	19	MCS	NA	NA

For 31 of the enrolled patients, the CRS-R evaluation could be performed weekly (mean interval between evaluations: 7.5 ± 2.9 days), which yielded a significant correlation between the number of CRS-R evaluations and the duration of patient care (correlation coefficient of 0.59 with p = 0.0005). Patients 5 and 6 could not be followed weekly, but they were still selected for the study since they satisfied the eligibility criteria. Our general results comparing classes 1 and 2 for each aforementioned characteristic are listed in Table 4. General patient information was matched for both classes 1 and 2 (p>0.05), particularly in terms of brain lesion localisation, brain damage aetiology, the number of CRS-R evaluations, the delay between the occurrence of brain lesions and first CRS-R/MBT assessment, and the delay between the occurrence of brain lesions and the last CRS-R assessment.

**Table 4:** Statistical characteristics of the patients emerging from DOC compared to the patients remaining in DOC. P-values corrected for multiple comparison with the Holm-Bonferroni method. ° statistical comparison between UWS patients and MCS patients having emerged from DOC, respectively. DOC: disorders of consciousness; UWS: unresponsive wakefulness syndrome; MCS: minimally conscious state; SD: standard deviation.

	Remaining in DOC	Emerging from DOC	p-value corrected
Demographic characteristics	n (%) or mean (SD)	n (%) or mean (SD)	
**DOC (n = 33)**	12 (36%)	21 (64%)	
** UWS (n = 20, 61%)**	5 (25%) in UWS	11 (55%)	>0.05°
	4 (20%) in MCS		
** MCS (n = 13, 39%)**	3 (23%) in MCS	10 (77%)	
	0 (0%) in UWS		
**Age (years)**	39.83 (15.5)	52.52 (17.6)	>0.05
**Number of CRS**	6.33 (2.1)	4.71 (2.33)	>0.05
**Delay brain injury—first CRS-R/MBT (days)**	13.5 (7.6)	9.85 (5.3)	>0.05
**Delay brain injury—last CRS-R (days)**	54.91 (11.7)	50.62 (24.5)	>0.05
**Rehabilitation duration (days)**	29.08 (11.2)	26.95 (15.9)	>0.05
**Lesion localization**			>0.05
** Frontal**	9 (75%)	12 (57%)	
** Temporal**	7 (58%)	9 (43%)	
** Parietal**	4 (33%)	12 (57%)	
** Occipital**	1 (8%)	3 (14%)	
** Basal Ganglia**	7 (58%)	5 (24%)	
** Midbrain**	4 (33%)	2 (10%)	
** Diffuse axonal injury**	1 (8%)	2 (10%)	
**Etiology**			>0.05
** Ischemic stroke**	3 (25%)	5 (24%)	
** Hemorrhagic stroke**	0	5 (24%)	
** Intraventricular hemorrhage**	0	1 (5%)	
** Traumatic brain injury**	5 (42%)	10 (48%)	
** Ruptured aneurism**	2 (17%)	2 (10%)	
** Anoxia**	2 (8.3%)	0	
** Infection**	2 (8.3%)	1 (5%)	
** Other vascular disease**	2 (8.3%)	1 (5%)	
**Secondary endpoint**			
** Functional walking ability**	3 (25%)	15 (70%)	0.021
** Return home**	3 (25%)	17 (81%)	0.007

Among the 33 enrolled patients, 20 patients (61%) were diagnosed as being in UWS, and 13 patients (39%) were diagnosed as being in a MCS at the first evaluation. Globally, 21 patients (64%) recovered consciousness (class 1); 12 patients (36%) remained with DOC (class 2). Specifically, 11 out of 20 UWS patients (55%) emerged from DOC (subclass (a)); among the UWS patients still in DOC (45%), 4 patients (20%) remained in UWS (subclass (c)) and 5 patients (25%) evolved to MCS (subclass (d)); among the MCS patients, 10 out of 13 patients (77%) emerged from DOC (subclass (b)), 3 patients (23%) remained in MCS (subclass (f)), and no patients worsened to UWS (subclass (e)). Although our outcome comparison between UWS and MCS patients was not statistically significant (p>0.05), the absolute values tended to be largely consistent with the data appearing in the literature [9, 15, 16]. However, there was no significant difference between TBI and NTBI, while recovery is expected to be more favourable in TBI [12, 14, 15]. Finally, the difference was significant for returning home (p = 0.007) and walking ability (p = 0.021).

Among the nine comparison analyses performed within each statistical step, four outcome comparisons exhibited significant differences and are discussed in detail below. Therefore, the results of the following subclasses’ comparisons are not discussed: For the UWS patients, both comparisons between subclass (d) and subclasses (a) or (d) because of their insignificant differences, and the three comparisons involving subclass (e) since no MCS patients worsened to UWS.

**Fig 1:**
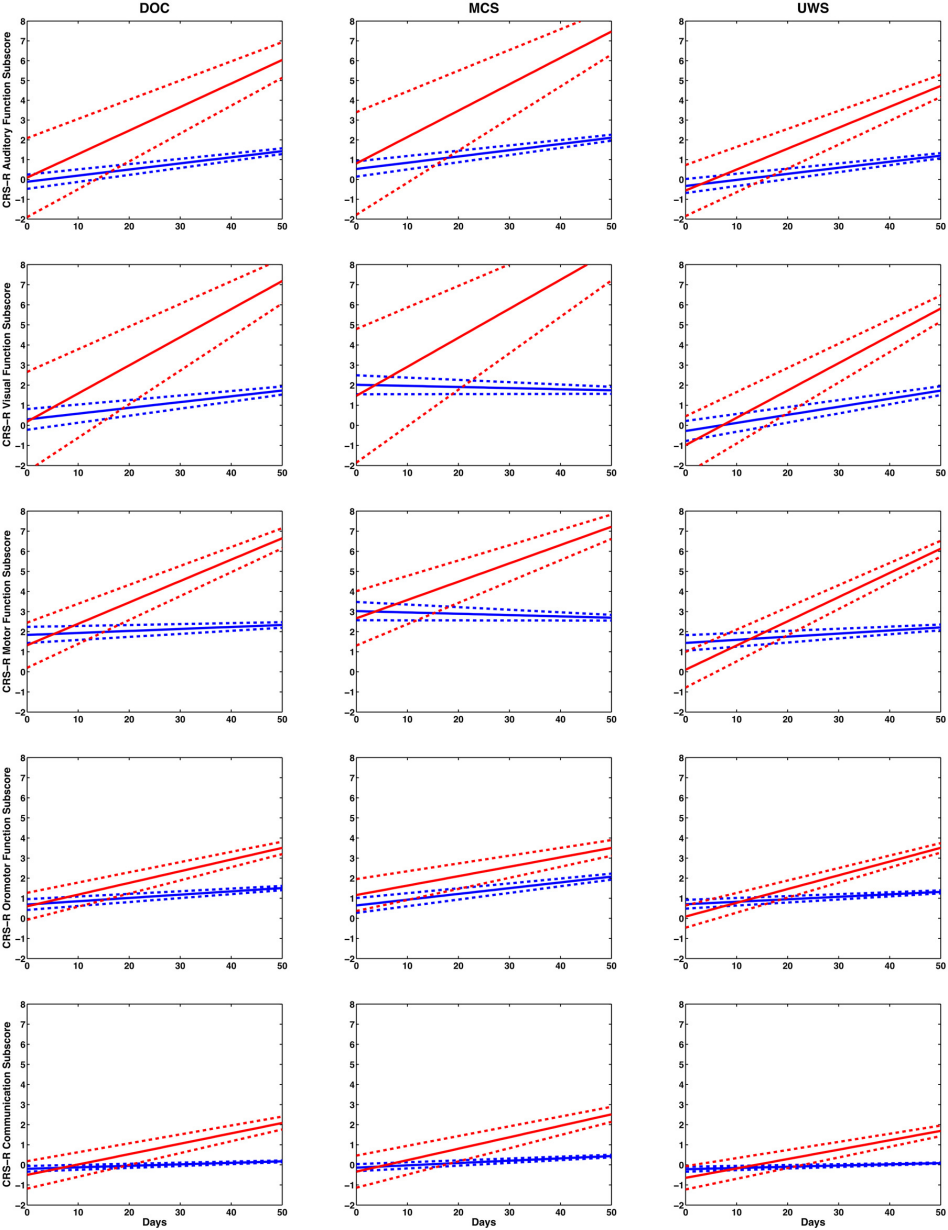
Fitting of the longitudinal CRS-R using the General Linear Modelling. GLM fitting of the longitudinal CRS-R subscales data; outcome differentiation over time for each subscale between patient emerging from DOC (in blue) and those remaining in DOC (in red); solid lines represent the mean of the time evolution and dash lines represent the superior and inferior bounds of the standard error of the means. Abscissa axe from top to bottom: auditory function scale, visual function scale, motor function scale, oro-motor function scale, communication scale; ordinate axe from left to right: DOC, UWS, MCS. Group distinction not earlier than 19 days according to the various subscales: 20 days for the auditory subscale for all groups of patients; 21 days for the visual subscale; between 19 and 21 days for the motor subscale; between 21 and 22 days for the oro-motor subscale; between 22 and 23 days for the communication subscale. Cut-off scores for the diagnosis of consciousness recovery reached after at least 29 days for the motor function subscale and after at least 40 days for the communication subscale. GLM: General Linear modelling; CRS-R: Coma Recovery Scale-Revised; DOC: disorders of consciousness; UWS: unresponsive wakefulness syndrome; MCS: minimally conscious state.

First, a statistical comparison of the first CRS-R subscales did not permit us to differentiate between the patients recovering consciousness (class 1) from those patients remaining with DOC (class 2) (Table 5). In the GLM analysis of the longitudinal CRS-R subscales, only combined together no-order (baseline) and first-order (slope) polynomial parameters, which outline the linear time course of the data, exhibited significant differences (p<0.02) when compared between both classes 1 and 2, between UWS subclass (a) and UWS subclasses (c) and (d) together, and between both MCS subclasses (b) and (f). Additionally, those time course differences yielded the following information: Temporal discrimination was not possible within the first 19 days for all subscales and the cut-off scores for the diagnosis of consciousness recovery were attained after 29 days for the motor function subscale and after 40 days for the communication subscale (Fig 1).

**Table 5:** Statistical prediction of the outcome from the CRS-R scores alone and weighted by the MBT scores (p-values corrected). Statistical prediction of the outcome from the first CRS-R subscores (auditory, visual, motor and oro-motor functions, and communication subscales) and from the same CRS-R subscores weighted by the MBT clinical values, assessed in the acute phase (mean 11.2 days after brain damage). Prediction proceeded for DOC classes, and for UWS and MCS subclasses. P-values corrected for multiple comparison with the Holm-Bonferroni method. CRS-R: Coma Recovery Scale-Revised; DOC: disorders of consciousness; UWS: unresponsive wakefulness syndrome; MCS: minimally conscious state; MBT: Motor Behavioural Tool.

	Auditory function subscale		Visual function subscale		Motor function subscale		Oro-motor function subscale		Communication subscale	
Comparison of patients	CRS-R subscore	CRS-R enhanced by MBT	CRS-R subscore	CRS-R enhanced by MBT	CRS-R subscore	CRS-R enhanced by MBT	CRS-R subscore	CRS-R enhanced by MBT	CRS-R subscore	CRS-R enhanced by MBT
**Remaining in DOC vs Emerging from DOC**	>0.05	0.008	>0.05	0.018	>0.05	0.018	>0.05	0.002	>0.05	0.002
**Remaining in UWS/MCS vs Emerging from DOC**	>0.05	0.007	>0.05	0.009	>0.05	0.004	>0.05	0.008	>0.05	0.009
**Remaining in UWS vs Emerging from DOC**	>0.05	0.007	>0.05	0.004	>0.05	0.007	>0.05	0.006	>0.05	0.003
**Remaining in MCS vs Emerging from DOC**	>0.05	>0.05	>0.05	>0.05	>0.05	>0.05	>0.05	>0.05	>0.05	>0.05

Second, a statistical comparison on the MBT values revealed that all positive (items 1–5) and negative (items 6 and 7) MBT signs yielded a significant predictive value for discriminating between classes 1 and 2; an exception was observed for item 7 in the comparison of both classes 1 and 2 (p>0.05). We also observed the same results for UWS patients when we compared subclass (a) with subclass (c), and with subclasses (c) and (d) together. Moreover, the three reflexes items (MBT 8–10) also presented predictive properties for overall DOC patients (a comparison of classes 1 and 2) and UWS patients (subclass (a) compared with subclasses (c) and (d) together). On the other hand, comparisons of other subclasses (comparison between the (b) and (f) MCS subclasses and between (a) and (d), and the (c) and (d) UWS subclasses) did not reveal statistically significant differences. The statistical comparison was characterised by elevated sensitivity, specificity, and Yule’s Q coefficient. Some sensitivity values were equal to 1, which was incompatible with the expected Bayes error rate (minimum error bound); this finding may be explained by the small sample size of some subclasses. The results are detailed in Table 6.

**Table 6:** Statistical prediction of the outcome from the MBT scores. Statistical prediction of the outcome from the clinical values of the MBT items, assessed in the acute phase at first CRS-R evaluation (mean 11.2 days after brain damage). Prediction proceeded for DOC classes, and UWS and MCS subclasses. Indices of the MBT items refer to the MBT indices presented in Table 1. P-values corrected for multiple comparison with the Holm-Bonferroni method. Sensitivity, specificity and Yule’s Q coefficient not assessed when p-values >.05 of the non-parametric Mann-Whitney U test. DOC: disorders of consciousness; UWS: unresponsive wakefulness syndrome; MCS: minimally conscious state; MBT: Motor Behavioural Tool.

	MBT 1	MBT 2	MBT 3	MBT 4	MBT 5	MBT 6	MBT 7	MBT 8	MBT 9	MBT 10
Comparisons of patients										
**Remaining in DOC vs Emerging from DOC**										
p-value (corr)	0.013	0.012	0.005	0.005	0.011	0.013	0.053	0.005	0.039	0.004
Sensitivity	0.86	0.81	0.90	<1	0.90	0.86	0.76	0.5	0.25	0.5
Specificity	0.67	0.75	0.67	0.5	0.58	0.67	0.58	<1	<1	<1
Yule's Q coef	0.91	0.91	0.92	0.91	0.9	0.91	0.84	0.91	0.75	0.91
**Remaining in UWS or MCS vs Emerging from DOC**										
p-value (corr)	0.019	0.016	0.019	0.017	0.03	0.015	0.03	0.03	0.04	0.03
Sensitivity	<1	0.91	<1	<1	0.91	<1	0.91	0.56	0.33	0.56
Specificity	0.67	0.78	0.67	0.67	0.67	0.67	0.67	<1	<1	<1
Yule's Q coef	0.91	0.92	0.91	0.91	0.88	0.91	0.88	0.86	0.69	0.86
**Remaining in MCS vs Emerging from DOC**										
p-value (corr)	>0.05	>0.05	>0.05	>0.05	>0.05	>0.05	>0.05	>0.05	>0.05	>0.05
Sensitivity	—	—	—	—	—	—	—	—	—	—
Specificity	—	—	—	—	—	—	—	—	—	—
Yule's Q coef	—	—	—	—	—	—	—	—	—	—

Third, a statistical comparison of CRS-R subscales weighted by the positive MBT signs allowed those subscales to become predictive for overall DOC outcome (class 1 compared with class 2) and for UWS patients ((a) subclass compared with (c) subclass, and with the (c) and (d) subclasses). Even if they had a low increment (0.5), all of the items of the positive MBT signs were particularly valuable for enriching all of the CRS-R subscales (p<0.019). On the other hand, differences between MCS subclasses (b) and (f), between UWS subclasses (a) and (d), and between UWS subclasses (c) and (d), were not statistically significant (Table 5).

The absence of significant differences between the MCS subclass compared with either the UWS subclass or with the non-DOC subclass can be explained by the small sample size and additionally by the closeness of the CRS-R subscores between these states.

Finally, VBM of brain lesions did not reveal any significant anatomical differences between classes 1 and 2 (p>0.05).

## Discussion

In this study, we systematically evaluated acute DOC patients using the CRS-R scheme and of our MBT; the latter has been designed to identify clinical signs of consciousness that the CRS-R scheme may overlook, thereby leading to DOC misdiagnoses by lowering the CRS-R score.

We present three primary findings:

1. A statistical comparison applied to the first CRS-R evaluation in the acute stage did not yield predictable properties, meaning that an accurate diagnosis and prognosis cannot be reliably attained during this critical period using only CRS-R scores. However, regression modelling on the CRS-R subscales enabled us to differentiate over time the patients remaining with DOC from those having emerged from DOC, but the outcome discrimination was not significant in the first 19–22 days, regardless of the CRS-R subscores. Moreover, we found that at least 29 days is required to attain the cut-off scores for the diagnosis of consciousness recovery given by the motor function and the communication subscales. Our data corroborate the understanding that the prognostic validity of the CRS-R remains unproven in the acute phase [30] [33] despite its good content validity [30] and its recommendation as a tool for establishing diagnosis and monitoring recovery of consciousness [31].
2. Our statistical comparison revealed that the positive items of the MBT help to better identify signs of consciousness and provide reliable predictability addressing consciousness recovery. All of those items check for slight motor activities, which are by definition overlooked by the CRS-R, which imposes strong criteria to consider a motor behaviour as the expression of consciousness. Concretely, the CRS-R does not support non-systematised hypokinetic movement, hieratic oculomotricity, the draft of intentional motor responses, and slight induced facial motricity by noxious stimulus or increased motor behaviour due to a motivational context. This leads to negative scoring, whereas such motor features may express a conscious activity, especially since concomitant motor or cognitive impairment hamper motricity [34]. The two negative MBT items also share predictive properties, in the sense of enlightening concomitant medical conditions, which offer a credible alternative to the explanation of impaired verbal or motor response. Finally, the presence of reflex behaviours speaks for increased consciousness impairment and poorer outcomes.
3. With regard to the reliable predictability of the positive MBT items, it became apparent that their introduction into the CRS-R assessment rendered the CRS-R subscores also significantly predictable, meaning that strong quoting rules do disservice to clinical evaluations of consciousness.

These results lead to two primary potentialities that should be discussed.

First, motor behaviour evaluation in the acute phase is a main way to accurately assess the content and the degree of consciousness and to predict outcome within the first 6 weeks. The fact that subtle motor behaviour can promote the predictability of DOC outcome, whereas the CRS-R subscores cannot, highlights the importance of exploring and considering all levels of motor activity that can reveal preserved consciousness. As a result, the CRS-R remains a valuable scheme that should be adapted to refine grading stages. With this finding in mind, additional studies should emphasise the performance analysis of all sub-items included in all the available evaluation schemes.

Second, our findings provide significant evidence that a reliable measure of conscious awareness in the acute phase necessarily needs to take into account the patient's capacity to perceive, understand and interact with his environment, his awareness of himself and his reaction to pain. Developed from evaluations starting as early as 24 hours after sedation withdrawal, MBT emphasises the observation of the broadest levels of subtle motor signs that may be seen as non-reflexive intentional responses in opposition to reflexive motor reaction; additionally, MBT takes into consideration concomitant medical conditions, such motor or communication disabilities, that hamper the patient's interaction with his environment [42]. The significant outcome prediction for both positive and negative MBT items, in terms of consciousness recovery and improvements in walking skills, may highlight the involvement of blocked motor efference rather than a true consciousness disorder. Patients presenting the "trap" of blocked motor efference might demonstrate the clinical signs of a new nosological entity: Cognitive motor dissociation [43], a condition in which “a lack of purposeful motor behaviour” hides motor response due to an underlying structural disruption between the motor cortex and the thalamus [44]. This condition leads to consciousness impairment overestimation, while the degree and level of consciousness are largely preserved. Establishing that a patient has such a neurological condition is of great significance with regard to therapeutic choices and global medical management because of a much better prognosis.

Additional improvements to MBT might investigate case-specific questions and address each patient individually by varying the testing conditions. Moreover, advances in neuroimaging technologies and the integration of electrophysiological paradigms into a reliable communication tool [45] might provide additional indications of awareness signs that ultimately lead to reducing the incidence of misdiagnoses and ensuring better treatment.

## Conclusion

The identification and classification of enlarged motor behaviour, without restrictions on their characteristics and the localisation of lesions along with their expected related symptoms, should be systematically considered to overcome the aforementioned limits of the existing scales. Such work would improve diagnostic and prognostic knowledge in DOC care in the acute phase.
